# 2,6-Diaminopyridine-Based Polyurea as an ORR Electrocatalyst of an Anion Exchange Membrane Fuel Cell

**DOI:** 10.3390/polym15040915

**Published:** 2023-02-11

**Authors:** Yen-Zen Wang, Tar-Hwa Hsieh, Yu-Chang Huang, Ko-Shan Ho

**Affiliations:** 1 Department of Chemical and Materials Engineering, National Yu-Lin University of Science & Technology, 123, Sec. 3, University Rd., Yun-Lin 64301, Taiwan; 2 Department of Chemical and Materials Engineering, National Kaohsiung University of Science and Technology, 415, Chien-Kuo Road, Kaohsiung 80782, Taiwan

**Keywords:** diaminopyridine, polyurea, cathode catalyst, AEMFC

## Abstract

In order to yield more Co(II), 2,6-diaminopyridine (DAP) was polymerized with 4,4-methylene diphenyl diisocyanates (MDI) in the presence of Co(II) to obtain a Co-complexed polyurea (Co-PUr). The obtained Co-PUr was calcined to become Co, N-doped carbon (Co–N–C) as the cathode catalyst of an anion exchange membrane fuel cell (AEMFC). High-resolution transmission electron microscopy (HR-TEM) of Co–N–C indicated many Co-Nx (Co covalent bonding with several nitrogen) units in the Co–N–C matrix. X-ray diffraction patterns showed that carbon and cobalt crystallized in the Co–N–C catalysts. The Raman spectra showed that the carbon matrix of Co–N–C became ordered with increased calcination temperature. The surface area (dominated by micropores) of Co–N–Cs also increased with the calcination temperature. The non-precious Co–N–C demonstrated comparable electrochemical properties (oxygen reduction reaction: ORR) to commercial precious Pt/C, such as high on-set and half-wave voltages, high limited reduction current density, and lower Tafel slope. The number of electrons transferred in the cathode was close to four, indicating complete ORR. The max. power density (P_max_) of the single cell with the Co–N–C cathode catalyst demonstrated a high value of 227.7 mWcm^−2^.

## 1. Introduction

Proton-exchanged membrane fuel cells (PEMFCs), which use acid electrolytes to transport protons, have durability problems caused by the corrosion of the metal parts of the membrane exchanged assembly (MEA) by the acid medium. The proton can be replaced with an anion such as hydroxy (OH^−^) to avoid acid corrosion if the electrolyte is not acid. Naturally, the electrochemical mechanism of the fuel cell would change, but the input gas fuel cell remains the same with H_2_ in the anode and O_2_ in the cathode. Additionally, the proton-transferring membrane cannot be Nafion and needs to be replaced with hydroxy-transporting polymers, which usually contain positive nitrogen (N^+^). This is called an anion exchange membrane fuel cell (AEMFC). Eventually, the redox reaction for an AEMFC would start from the cathode, where O_2_ is reduced to become OH^−^ after combining with water, which is still the bottleneck reaction for the entire redox reaction.

To improve the oxygen reduction reaction (ORR) in the cathode, many metal–organic frameworks (MOFs) act as catalysts. The captured (complexed) transitional metal behaves as the catalytic center to absorb O_2_ for ORR. However, the framework needs to conduct the electrons from the anode through the external circuit. In other words, we can use an electron-conducting MOF (EC-MOF) to catalyze the ORR in the cathode. To enhance the O_2_ absorption capability by increasing the polarity of the carbon-dominated framework, the metal is usually coordinated with several nitrogen atoms and is calcined to form the so-called metal, nitrogen-doped carbon network (M–Nx–C) as the ORR catalyst. Melamine is usually calcined to become M–N–C catalysts in the presence of metal ions. However, melamine can be complexed firmly with metal ions since it is not a cyclic compound that can enclose the metal inside. A long molecule (polymer) cannot entangle metal ions.

Consequently, we need to gradually increase the size of polymelamines to capture the metal ions by several reaction steps [1,2,3,4]. Melamine needs to combine with other conducting polymers [5] or matrix [6,7,8,9] before calcining to become M–Nx–C catalysts.

Another option is to use a macrocyclic compound [10,11,12,13,14,15] to capture metal ions by nitrogen, i.e., N MOFs. Two major N-macrocyclic compounds used to prepare MOFs and M–Nx–Cs are porphyrin [16,17,18,19,20,21] and phthalocyanine [22,23,24,25,26,27,28,29]. Both use four -Ns to complex with the central metal and can create an M–N4–C structure after calcination. Although MOFs can quickly create an M–Nx–C structure after calcination, they are still individual units that are not connected. Furthermore, they will release the captured metal during calcination if decomposition occurs. Therefore, an additional carbon matrix [6,7,8,9] is needed to adhere to the MOF unit to prevent the collapse of the cyclic structures.

To avoid the trouble of preparing the macrocyclic structure and the addition of a carbon matrix such as carbon black (CB), we have prepared many nitrogen-containing polymers (N polymers). The N polymers can chelate with cobalt ions (Co(II)) and calcine to create a Co–N–C structure [30,31,32,33] without the addition of any CB.

DAP has a similar structure as melamine, except it is bifunctional, not trifunctional. However, its reactivity is higher than that of melamine and can be polymerized by either addition or condensation. It can be polymerized by free-radical polymerization using persulfate salts as the initiator in the aqueous solution to obtain poly(2,6-diaminopyridine) (PDAP). Pyridine’s strong complexing capability can be applied to extract toxic metal ions for environmental purposes [34]. When it is polymerized with dialdehyde via condensation, it becomes a catalyst that can absorb CO_2_ to ignite the reduction reaction [35,36]. DAP alone can implant on the graphene oxide surface to enhance the capture of dopamine [37].

To increase the number of nitrogen in the N-polymers and to allow the nitrogen to be close to the Co(II), we replaced 4,4-phenylene diamine (PDA) with 2,6-diaminopyridine (DAP) to prepare Co(II)-complexed polyurea (Co-PUr) by condensate with 4,4-diphenyl methylene diisocyanate (MDI). Eventually, we had three nitrogen atoms located at the sides of each monomer unit of Co-PUr. The Co-PUr will go through two stages of calcination performed in argon and ammonia. Between the two steps of calcination, there is the acid-washing procedure to remove the magnetic products. The obtained Co–N–Cs were characterized by X-ray diffraction (XRD), energy dispersive X-ray spectroscopy (EDs), X-ray photoelectronic spectroscopy (XPS), and electron microscopy. The electrochemical properties of the Co–N–C catalysts were measured by current-potential polarization (C–V) and linear sweeping voltammetric (LSV) curves. An MEA based on the cathode catalysts of Co–N–Cs was fabricated and assembled into a single cell to measure the polarization curve and obtain the cell’s power density.

## 2. Materials and Methods

### 2.1. Preparation

#### 2.1.1. Preparation of Co-PUr (The Catalyst Precursor)

**Scheme 1:**
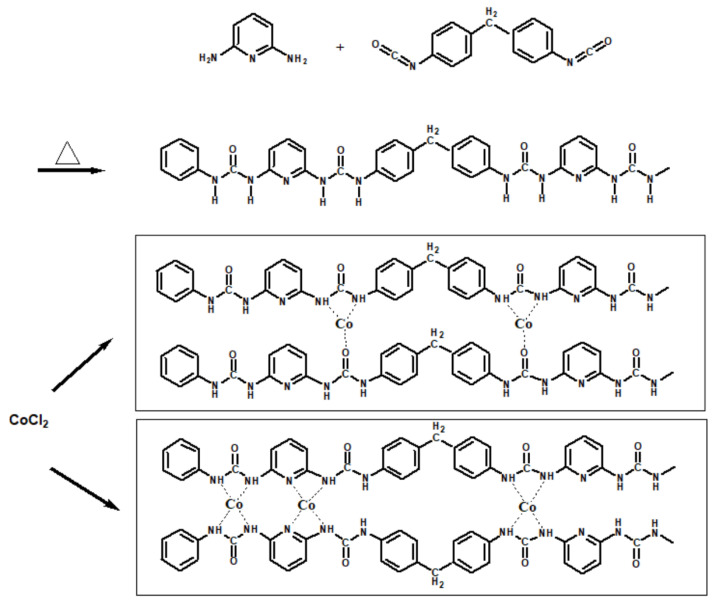
Schematic diagram of the polymerization and Co(II) chelation of PUr.

MDI (2.5 g, 98%: Acros, Fukuoka, Japan) and 1.635 g of DAP (98%, Tokyo Kasei Kogyo Co., Ltd., Tokyo, Japan) were uniformly dissolved in 30 mL methyl ethyl ketone (MEK, 99%: ECHO Chemical Co., Ltd., Kaohsiung, Taiwan). After the mixture solution was clear, we placed it on a heating plate and started heating. During the heating period, the solution was continuously stirred. When the solution temperature reached 70 °C, a polymerization reaction occurred. After stirring for 30 min, 0.645 g of anhydrous cobalt chloride (CoCl_2_, J.T. ker, Radnor, PA, USA) dissolved in 50 mL acetone (99%: ECHO Chemical Co., Ltd., Kaohsiung, Taiwan) was uniformly poured into the polymerization mixture. The solvent was completely evaporated at 80 °C, and the residue product was dried in an oven for 10 h to obtain Co-PUr. Polyurea prepared without Co(II) was prepared for comparison and named PUr. The preparation is described in Scheme 1.

#### 2.1.2. Preparation of Co–N–Cs

For this preparation, we weighed 0.5 g of Co-PUr and carried out the first calcination in a quartz boat; the calcining temperature was set to 900 °C (or 800 or 700 °C) at 10 °C per min. When the temperature reached 900 °C (800 or 700 °C), nitrogen was introduced into the tubular furnace and calcination was maintained for 1 h. After the temperature of the furnace was lowered to room temperature, the first calcination sample was obtained. In order to remove the formed magnetic cobalt oxide, the calcined sample was ground into powder before immersion in 9 M H_2_SO_4_ (95–97%, Honeywell, NJ, USA) at 65 °C for 24 h. In order to avoid corrosion through the filter paper, the 9 M H_2_SO_4_ was diluted to 1 M before filtration to obtain a filter residue. The filter cake was washed with acetone and deionized water and placed in a petri dish and then dried in an oven at 80 °C for 10 h. Then, the sample was calcined at 100 °C lower than that of the first calcination. It was re-calcined at 800 °C (700 °C or 600 °C) in mixed gases of equal volumes of ammonia and nitrogen for 1 h. After the furnace temperature dropped to room temperature, it was dried in an oven set at 80 °C for 10 h, and the Co–N–Cs with different histories were obtained.

### 2.2. Characterization

#### 2.2.1. Fourier-Transform Infrared (FTIR) Spectroscopy

The functional groups of PUr and Co-PUr were characterized by FTIR spectroscopy. The FTIR spectra were recorded on an IFS3000 v/s Fourier-transform infrared spectrometer (Bruker, Ettlingen, Germany) at room temperature.

#### 2.2.2. Scanning Electron Microscopy (SEM)

Images of all Co–N–Cs were captured using a field-emission SEM (AURIGAFE, Zeiss, Oberkochen, Germany) prepared from strewn-on carbonic tape.

#### 2.2.3. High-Resolution Transmission Electron Microscopy (HR-TEM)

Samples for the field emission transmission electron microscopy, HR-AEM (HITACHI FE-2000, Hitachi, Tokyo, Japan) were first dispersed in acetone and then placed on carbonic-coated copper grids drop wise before being subjected to the emission.

#### 2.2.4. X-ray Diffraction Spectroscopy (XRD)

A copper target (Cu-Kα) Rigaku X-ray source (Rigaku, Tokyo, Japan) with a wave-length of 1.5402 Å was used for diffraction. The scanning angle (2θ) ranged from 5° to 70° with a voltage of 40 kV and a current of 30 mA at 1 degree min^−1^.

#### 2.2.5. X-ray Photoelectronic Spectroscopy (XPS)

The various binding energy spectra of Co2p of CoNC-900A800 were used to characterize the percentage of cobalt in the form of Co0, CoNx, and CoOx after calcination in an XPS instrument assembled by Fison (VG) -Escalab 210 (Fison, Glasgow, UK) using a Al Kα X-ray source at 1486.6 eV. The pressure in the chamber was kept at 10^−6^ Pa or less during measurements. The binding energies of the Co2p around 780 eV were recorded.

#### 2.2.6. Raman Spectroscopy

The Raman spectra of various Co–N–Cs were carried out by a Triax 550 spectroscopy (HOBRIA, Kyoto, Japan) with a green laser light source of 520 nm. The samples were untreated before exposure to the Raman source.

#### 2.2.7. Energy Dispersive X-ray Spectroscopy (EDs)

The EDs spectra of the Co–N–Cs were obtained from an XL-40EFG by Philips (Amsterdam, The Netherlands). The samples were coated with gold before measurement.

#### 2.2.8. Brunauer–Emmett–Teller (BET) Analysis

Nitrogen adsorption–desorption isotherms are measured with an Autosorb IQ gas sorption analyzer (Micromeritics-ASAP2020, Norcross, GA, USA) at 25 °C. All of the trapped gas inside Co–N–Cs was removed at a temperature higher than 100 °C overnight. The surface area was obtained based on the BET equation when a linear BET plot with a positive C value was in the relative pressure range. Pore size distribution was determined by the Quenched Solid Density Functional Theory (QSDFT) method based on a slit/cylinder pore model. The total pore volume was determined at P/P_0_ = 0.95.

#### 2.2.9. Electrochemical Measurements

##### Current–Voltage (C–V) and Linear Sweep Voltammetry (LSV) Curves

Firstly, 0.029 g of catalyst was dispersed in a mixed solvent of 357 μL of ethanol and 357 μL of deionized water, and 20% Nafion solution was added as a dispersant and adhesive. The catalyst mixture was shaken for 24 h using an ultrasonic oscillator to become a catalyst slurry. Five μL of the slurry was evenly spread on the glassy carbon electrode, which was placed in an oven at 60 °C. Then, the glassy carbon electrode was installed on the rotating electrode of the potentiostat after it dried completely. We used a Ag/AgCl reference electrode and platinum wire as the counter electrode. Both electrodes were placed in 200 mL of 0.1 M KOH (99% SHOWA, Tokyo, Japan) electrolyte. Before the measurement, the KOH electrolyte was charged with O_2_ for 30 min, the rotation speed of the rotating disk electrode (RDE: Metrohm, Herisau, Switzerland) was adjusted, and the LSV curves were obtained. The C–V curve was obtained with the same procedures except that the electrode was static.

##### Membrane Electrode Assembly (MEA)

For MEA, 0.018 g each of the Co–N–C catalyst and Pt/C was added to 0.4 g DI water, 0.4 g methanol, and 0.09 g XB-7 (alkaline ionomer solution) (Dioxide Materials, Boca Raton, FL, USA) in sequence, shaking the vials for 24 h in an ultrasonic oscillator to uniformly disperse the catalyst in the slurry. We cut the carbon paper (N1S1007) into an appropriate area, placed it on the heating plate, and then spread the catalyst slurry uniformly on the carbon paper. The Pt/C catalyst slurry was prepared in the same way. The carbon papers containing Co–N–C and Pt/C and the X37-50RT (Dioxide Materials, Boca Raton, FL, USA) anion exchange membrane (AEM) were soaked in 0.1 M KOH for 24 h. After soaking, the residual KOH was cleaned with DI water. Finally, the carbon papers were sandwiched on both sides of the AEM and hot-pressed at 140 °C with a pressure force of 70 kgf cm^−2^ for 5 min to obtain the MEA.

##### Single Cell Testing

The MEA was then assembled on a testing station for the fuel cell to obtain the exporting potentials and power densities of the single cell with increasing current densities through a testing device (model FCED-P50; Asia Pacific Fuel Cell Technologies, Ltd., Miaoli, Taiwan). The performance cell area was 2 × 2 cm. The temperatures of the entire testing station including the anode, cell, cathode, and humidifying gas were kept at 60 °C. The flow rates of the H_2_ (anode) and the O_2_ (cathode) were controlled at 30 and 60 mL·min^−1^, respectively, in accordance with stoichiometry.

## 3. Results and Discussion

### 3.1. FTIR Spectra

**Figure 1:**
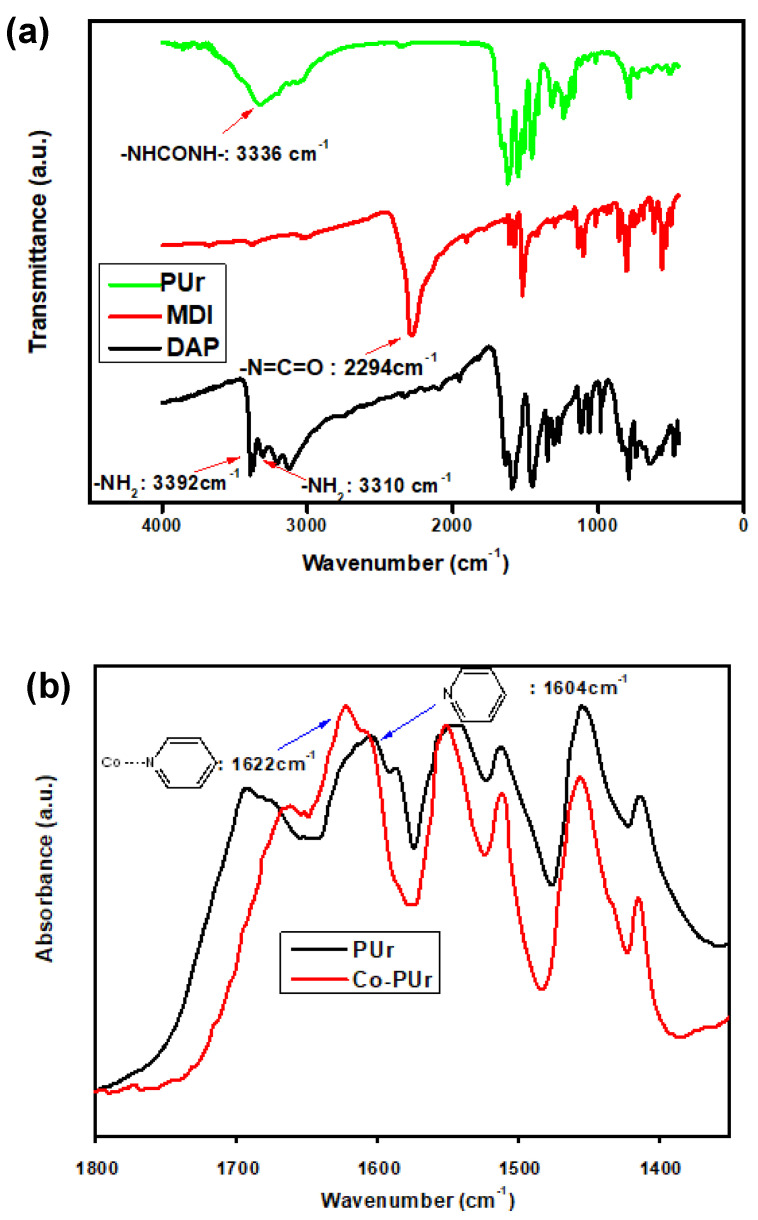
IR spectra of (**a**) PUr and (**b**) Co−PUr.

The isocyanate group of MDI demonstrates a pronounced absorption peak at 2294 cm^−1^. After the polymerization with DAP to form PUr, the absorption peak disappears, as seen in Figure 1a. In addition, the peak of primary amine belonging to DAP has two absorption peaks at 3385 and 3310 cm^−1^ (symmetric and asymmetric stretches) [38]. These two peaks merge into one (3336 cm^−1^) after polymerization, as seen in Figure 1a. The primary amine has already become a secondary amine when it converts to urea after reacting with the isocyanate group of MDI. All of the results can prove the successful synthesis of PUrs.

**Figure 2:**
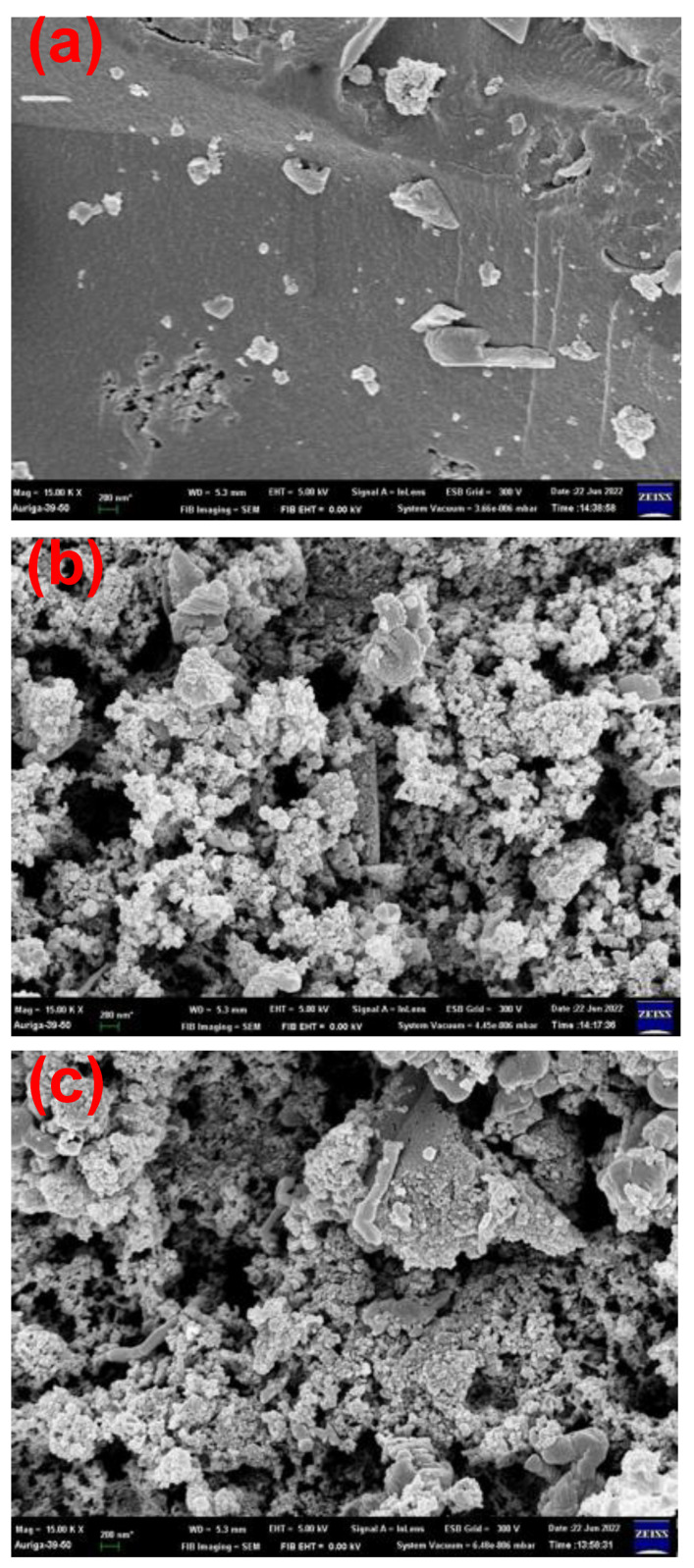
SEM micropictures of (**a**) CoNC-900A800 (**b**) CoNC-800A700 (**c**) CoNC-700A600.

The pyridinic nitrogen (py-N) can be expressed in the -C=N- of the aromatic ring (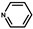), which contributes to a stretch band at ca. 1604 cm^−1^ in the IR spectrum of uncomplex, neat PUr, as seen in Figure 2b. After chelating with Co(II) (Co-PUr), the peak splits into two peaks at 1622 and 1604 cm^−1^ (demonstrating a shoulder form) following Figure 1b [39]. In other words, introducing the pyridine group into the polymer backbone can effectively increase the number of complexed Co(II).

### 3.2. SEM Micropictures

The purpose of carrying out calcination of Co-PUrs in the presence of N_2_ and NH_3_ (especially NH_3_), which can also effectively bombard the surface of catalysts to form more pores (except for compensating the loss of N atoms), is to create micro- or mesopores on the surfaces of the CoNC products. Both micro- and mesopores can accommodate the fuel O_2_ molecules and ignite ORR. The urea groups (-NH-CO-NH-) that contain both oxygen and hydrogen atoms can release water to form carbodiimide (-N=C=N-) during high-temperature calcination. We can see apparent inflation of the calcining samples when the temperature exceeds 700 °C, which is likely attributable to the release of water vapor. The evolution and evaporation of water can also create more pores on the surface of Co-M-Cs.

The SEM pictures of Co-M-Cs illustrated in Figure 2 reveal the surface morphologies of samples treated with different calcination temperatures. For CoNC-700A600, no significant pores are seen on the smooth surface (Figure 2a), indicating the calcination temperature is not high enough for either N_2_ or NH_3_ to excavate any holes on the surfaces. When the temperature exceeds 700 °C (CoNC-800A700 and -900A800), the surface roughness significantly increases due to the appearance of either micro- or mesopores, as seen in Figure 2b,c. We abruptly increased the BET surface areas for both the CoNC-800A700 and -900A800 samples, which will be discussed in the BET section.

### 3.3. HR-TEM Micropictures

**Figure 3:**
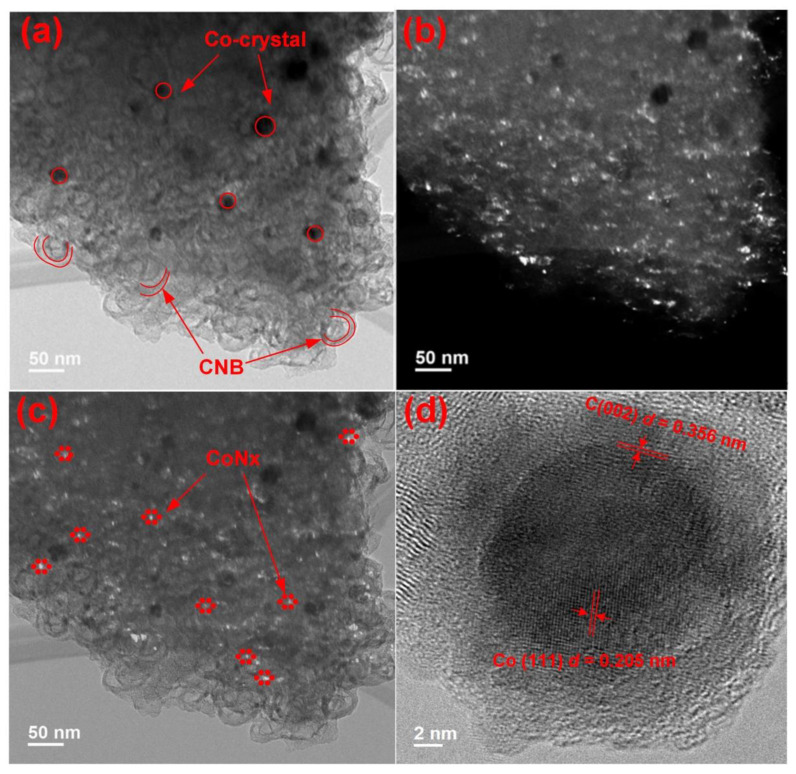
HR-TEM micropictures of CoNC-900A800 (**a**) bright field (**b**) dark field (**c**) overlapping of bright and dark fields (**d**) higher resolution picture for Co crystal.

The presence of the CoNx centers can be revealed by comparing the micropictures taken in the bright and dark fields of HR-TEM as seen in Figure 3a,b, respectively. In the bright-field picture (Figure 3a), the giant Co crystals are randomly distributed in the carbon matrix, which is full of carbon nanobelts (CNBs) or carbon nanorings (CNRs). Obviously, the Co atoms behave as the seeds to grow CNB or CNR during high-temperature calcination. The nanobelt or nanoring grows by adding each carbon shell one on one, starting from the crystallized Co seeds [39,40,41,42,43]. However, there is no evidence of the existence of the CoNx center in the bright-field picture demonstrated in Figure 3a. Considering the capability to reflect electrons for metal, a picture taken in the dark field is used to illustrate the shining dots of the Co-active centers, as shown in Figure 3b. Many tiny shining spots in the Co–N–C matrix (CoNC-900A800) reveal the formation of many CoNx centers after calcination. To clearly locate the position of the CoNx centers, we eclipse the bright and dark pictures in Figure 3c, which showed that most of the CoNx centers are not located within CNBs or CNRs but stay out of them. This indicates that CNB and CNR did not originate from CoNx centers but from the Co crystals. The large dark particles in Figure 3c are the crystallized Co elements created beneath the carbon matrix during calcination. The HR-TEM picture of one of the particles is illustrated in Figure 3d, demonstrating the clear Co(111) plane with a d-spacing of 0.205 nm (2θ = 44°) inside the particle. Furthermore, on the brink and surrounding area of the particle, we can observe the C(002) plane of the carbon matrix, and the d-spacing is ca. 0.356 nm, which can be obtained from the diffraction peak at 25° (2θ) illustrated in the XRD section.

### 3.4. XRD Pattern

**Figure 4:**
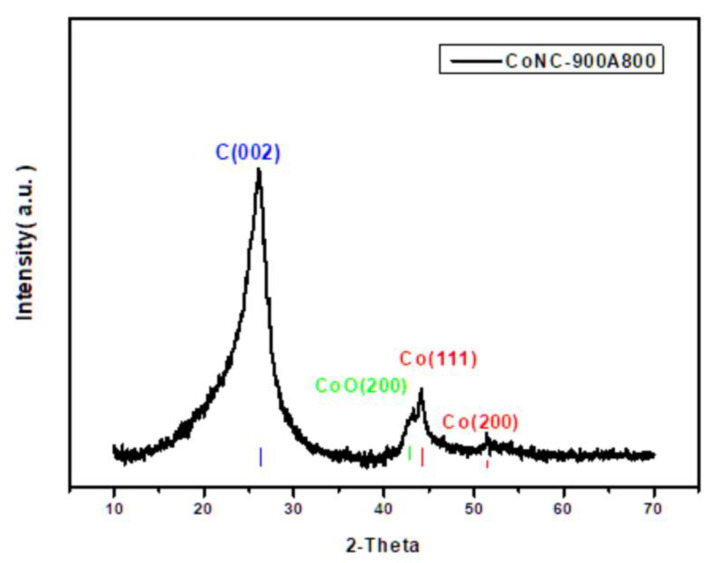
X-ray diffraction pattern of CoNC-900A800.

CoNC-900A800 was chosen to analyze the ordered crystalline structure of CoNCs by XRD patterns. The pattern displayed a significant peak at about 25° related to the C(002) plane, as seen in Figure 4 for CoNC-900A800. The remaining peaks exhibited smaller intensities at 2θ = 43°, 44°, and 51.5°, corresponding to the CoO(200), Co(111), and Co(200) planes of crystallized cobalt, respectively. Based on Bragg’s law, the d-spacings for the C(002) and Co(111) planes are 0.356 and 0.205 nm, respectively, matching the results obtained from the HR-TEM picture in Figure 3d. The affluent crystallized carbon composition of carbon nanobelts (CNBs) or carbon nanorings (CNRs) indicates that a highly conductive carbon matrix is ready to serve as the medium for the electrons from the anode. The unprotected Co crystals or Co oxides (CoO) can be removed by acid leaching. Therefore, the Co-crystalline formed is believed to be under the protection of the carbon matrix and is free from oxidation during acid leaching and calcination. Co crystals can explain why many CNBs and CNRs were created during calcination. As seen in the TEM pictures, the Co (or Fe) crystal acts as the seed to grow CNTs from consuming other carbon matrices [41,42,43,44,45,46].

### 3.5. XPS

**Figure 5:**
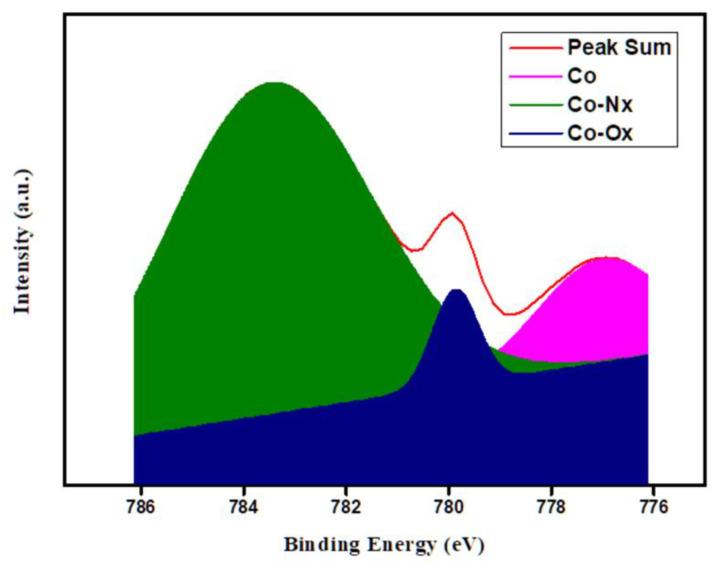
XPS-Co2p of CoNC-900A800.

The Co(II)-complexed PUr can induce several reactions such as reduction, oxidation, and nitridation during calcination. The obtained Co–N–C catalysts (CoNC-900A800 as the example) are mostly made of Co0, Co-Ox, and Co-Nx, which the Co2p of the XPS can measure, as seen in Figure 5. The Co0 created by thermally induced reduction on the catalyst surface can be removed by acid leaching. Most of the Co-Ox, contributing to the magnetic activity, can also be neutralized and removed by 9 M H_2_SO_4_. Therefore, only Co-Nx, which constitutes the framework of the Co–N–C catalyst, remains. The XPS of Co2p of CoNC-900A800 shown in Figure 5 reveals that Co-Nx is the dominant component of the catalyst. However, we still found some Co0 and Co-Ox, which were not removed by acid leaching.

### 3.6. Raman Spectroscopy

**Figure 6:**
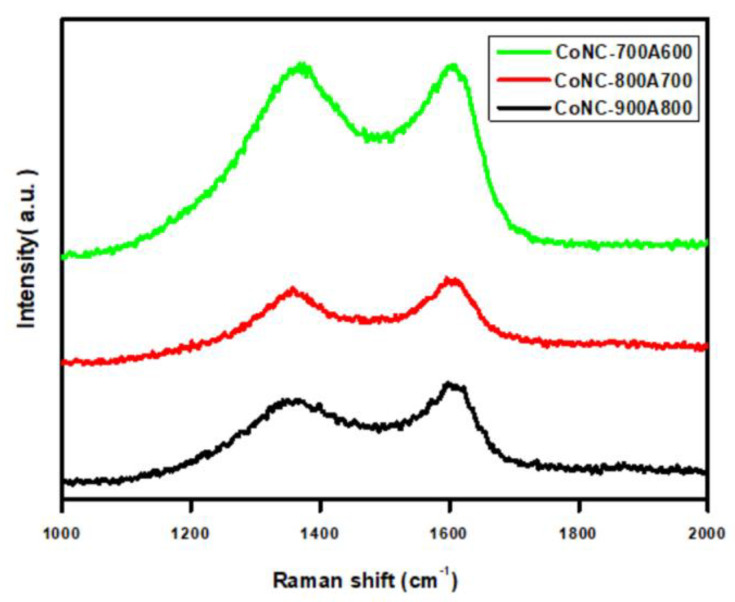
Raman spectra of (black) CoNC−900A800 (red) CoNC−800A700 (green) CoNC−700A600.

The Co-N-doped carbon structures in the Co–N–Cs were studied by Raman spectroscopy. Similar to common calcined carbon materials, two peaks with close height were seen at around 1350 and 1600 cm^−1^ for every Co–N–C catalyst, called the D- and G-band, respectively. After separating the peaks belonging to the D- and G-bands by performing deconvolution of the Raman spectra in Figure 6, the intensity ratios of the two peaks (I_D_/I_G_) were calculated for all catalysts and are listed in Table 1. The results demonstrate that the I_D_/I_G_ value is almost independent of the calcination conditions (temperature and acid leaching). However, slight structural ordering can be seen from the decreasing values of the I_D_/I_G_ ratios with increasing calcination temperature. When the I_D_/I_G_ ratio is less than one for CoNC-900A800, the number of sp^2^ carbons surpasses that of sp^3^. The ordering structure with calcining temperature originates from the development of the CNT and GF carbon structures, contributed from the increasing numbers of sp^2^ carbons (G-band) as shown in the XRD pattern of CoNC-900A800 in Figure 4 and will be seen in the TEM pictures demonstrated in the subsequent discussion.

### 3.7. EDs

**Figure 7:**
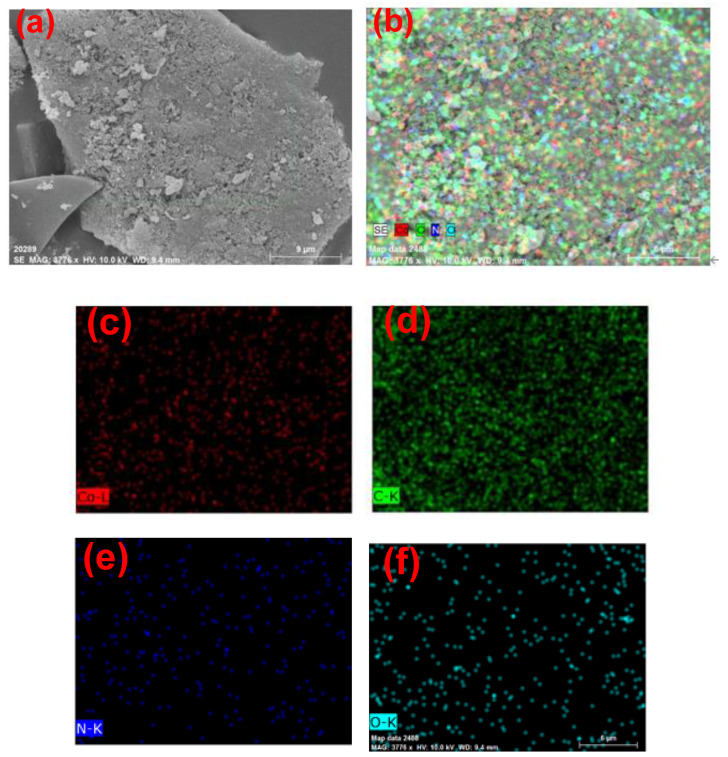
(**a**) SEM picture (**b**) EDs (**c**) cobalt (**d**) carbon (**e**) nitrogen (**f**) oxygen mappings of CoNC-900A800.

The SEM picture and EDs of CoNC-900A800 are illustrated in Figure 7a,b, respectively. The dispersions of Co, N, C, and O elements on the CoNC-900A800 surface can be observed from the mapping diagrams (Figure 7c–f), which are uniformly distributed on the catalyst surface. Table 2 shows the analysis of the compositions of all Co–N–C catalysts. The increase in the C element content indicates the increase in the degree of the catalysts^,^ graphitization, which can improve electron conduction. High-temperature calcination can reduce the content of the oxygen element, improving the conductivity of the catalyst. The presence of nitrogen is beneficial for the Co-active site of the catalyst. However, high-temperature calcination will lose some of the N. The secondary calcination performed on the acid-leaching catalysts can increase the number of pores on the catalyst and supply more N to the catalyst surface.

### 3.8. BET Surface Area and Pore Size

**Figure 8:**
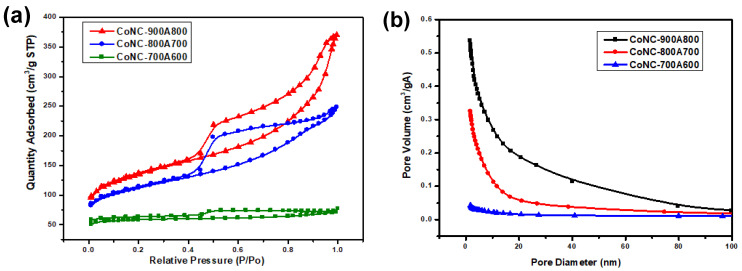
BET analysis of various Co–N–C catalysts. (**a**) N_2_ adsorption–desorption isotherms and (**b**) pore size distribution.

Figure 8a shows the catalysts^,^ nitrogen adsorption and desorption curves at different calcination temperatures. Catalysts with different end-calcination temperatures belong to the type (IV) adsorption isotherm curve and the third type of hysteresis curve in the IUPAC isotherm classification. The BET surface area (BSA) of CoNC-700A600 is not high and is still below 200 m^2^g^−1^ (177.74 m^2^g^−1^). The BSA jumped more than 300 m^2^g^−1^ (369.43 m^2^g^−1^) after the treated temperature increased from 700A600 to 800A700. Eventually, the BSA was 447.81 m^2^g^−1^ for CoNC-900A800. According to Figure 8a and Table 3, high-temperature calcination can increase the specific surface area of the catalyst, expose the CoNx-active sites of the catalysts to the O_2_, and be more capable of carrying out the ORR.

The pore size distribution is shown in Figure 8b, and the average pore sizes of the Co–N–C catalysts at different calcination temperatures are listed in Table 3. Most of the created pores are microporous, as shown in Figure 8a and Table 3. More Co crystals and Co- oxides (CoO) accumulated with increasing calcination temperature. Micropores remained on the catalyst’s surface when they were washed away by acid leaching. The number and the size of pores of the catalyst can increase by passing through ammonia gas in the second calcination to improve the ORR.

### 3.9. Electrochemical Measurements

#### 3.9.1. C–V and LSV Curves

We demonstrated in Equation (1) that one O_2_ molecule and one water molecule can be fully reduced to four hydroxyl ions (OH^−^) by cathode catalysts in an alkaline solution, following the 4e-pathway. However, HO_2_^−^ is an unavoidable intermediate by-product [30,31,32,33,40] due to the incomplete ORR (oxygen reduction reaction) in an alkaline medium (Equation (2)). Based on Equation (2), the reduction reaction can continue further after the supply of two additional electrons, the so-called 2e-pathway (Equation (2) plus Equation (3)). In other words, the four electrons from the anode are consumed in two separate steps, not one step. Unfortunately, hydroxyl radicals (OH^.^) may evolve from the cleavage of hydrogen peroxide ions (HO_2_^−^) following the one-electron reduction reaction (Equation (4)). The presence of OH· can prompt the degradation of the ionomer solid-state electrolyte membrane and the loss (oxidation) of CoNx- active centers to CoO (Equation (5)).

O_2_ + 4e^−^ + H_2_O → 4OH^−^ (1)

O_2_ + 2e^−^ + H_2_O → HO_2_^−^ + OH^−^ (2)

HO_2_^−^ + 2e^−^ → 3OH^−^ (3)

OH^−^+ e^−^ → HO· + O^2−^ (4)

Co(II) + O^−2^ → CoO (5)

The Co–N–C catalyst is made up of Co atoms coordinated with several N atoms (CoNx-) and implanted randomly on a N-doped carbon matrix. The presence of N–C bonding can increase the polarity of the carbon matrix. The electrocatalytic activity of the N–C bond originates from the electronegative N compared to C, resulting in polarized carbon. Consequently, the polarity of the carbons that are covalently bonded to Ns is increased to adsorb O_2_. ORR begins with the adsorption of O_2_ by the active centers (CoNx-), followed by the opening of the O=O bond. Very possibly, only one of the bonds (π-bond) of the O_2_ is opened due to its lower activation energy or shortage of electrons (incomplete ORR), resulting in a peroxide product such as HO_2_^−^ (Equation (1)). Thus, the peroxide product can cause damage to the organic electrolyte membrane and carbon structure of the cathode catalyst (Equations (4) and (5)) during the reduction reaction.

**Figure 9:**
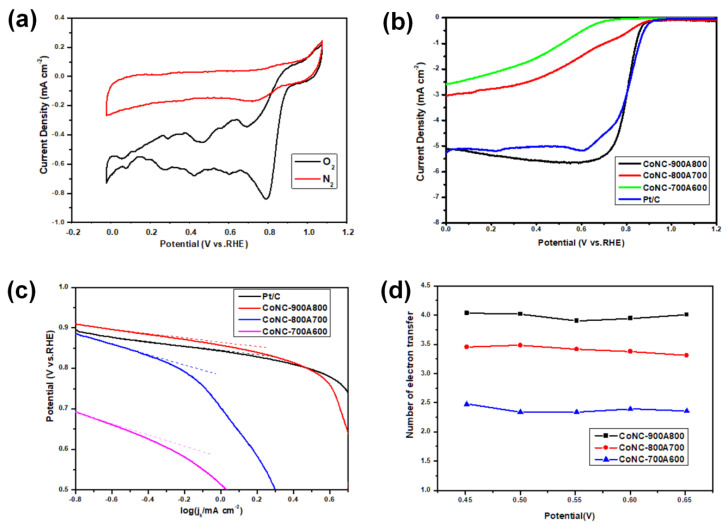
(**a**) C–V curve of CoNC−1000A900 conducted in O_2_ and N_2_ saturated, 0.1 M KOH_(aq)_, (**b**) LSV curves obtained at 1600 rpm in O_2_-saturated, 0.1 M KOH_(aq)_, (**c**) Tafel slopes of various Co–N–C catalysts, and (**d**) e-transferred values of various Co–N–C cathode catalysts.

The C–V polarization curve in Figure 9a demonstrates a significant reduction peak of ORR at ca. 0.8 V in the O_2_-saturated 0.1 M KOH(aq) electrolyte, which is not found when O_2_ is replaced by N_2_, revealing the high selectivity of the CoNC-900A800 catalyst.

The reduction current is recorded vs. the applied voltage in Figure 9b with the rotating disk electrode (RDE) setting at various rpms, and LSV curves are obtained for various Co–N–Cs in the O_2_-saturated 0.1 M KOH(aq) electrolyte to study the ORR. The on-set voltage (OSV), which is related to the ORR behavior of the cathode catalyst, is only 0.78 V for CoNC-700A600. After the calcination temperature increased, the OSV becomes 0.85 and 0.88 V for CoNC-800A700 and CoNC-900A800, respectively, comparable to that of a commercial Pt/C catalyst (0.89 V), as seen in Table 4. Another property concerning the ORR is the half-wave voltage (HWV), i.e., the mid-way of the kinetic-controlled ORR. CoNC-800A700 and -700A600 demonstrate low values (0.41 and 0.39 V, respectively), compared with Pt/C (0.83 V). However, the HWV is significantly improved for CoNC-900A800 (0.82 V) and is very close to Pt/C. This indicates that a higher calcination temperature can obtain either higher OSV or HWV. The Co-PUr precursor completely degrades if it is calcined at 1000 °C, which is why the maximum calcination temperature is maintained at 900 °C.

The LSV curve for each catalyst is constructed vs. voltage, from which the Tafel slope is obtained and shown in Figure 9c. A smaller Tafel slope means the reduction current drops very quickly with increasing potential. In other words, more ORR activity is retained. The Tafel slopes of CoNC-800A700 and -700A600 (165.9 and 164.1 mV dec^−1^, respectively) exceed 150 mV dec^−1^, and that of CoNC-900A800 is less than 100 mV dec^−1^ (66.2 mV dec^−1^), which is very close to that of Pt/C (67.9 mV dec^−1^), illustrated in Table 4. In other words, CoNC-900A800 and Pt/C have more active oxygen reduction reactivity than CoNC-800A700 or -700A600. This implies the necessity of increasing the calcination to 1000 °C, which can create more active sites and surface area for the Co–N–C catalysts.

The kinetic reducing currents obtained from RDE at various rpms were used to construct the Koutecký–Levich (K-L) lines and calculate the slopes as the average number of electrons transferred (e-transferred) by the Koutecký–Levich equation, as shown in Figure 9d and Table 4. CoNC-900A800 demonstrates an almost complete ORR with an e-transferred value approaching four, compared to CoNC-800A700 (3.5) or -700A600 (2.5). As discussed in the HR-TEM section, the two main ORRs are the 4e- (Equation (1)) and 2e-pathways (Equations (2) and (3)). CONC-900A800 can fully catalyze the ORR in the 4e-pathway without the evolution of the hydrogen peroxide ion (HO_2_^−^). However, 25% of the 2e-pathway occurs for the CoNC-800A700 catalyst when the total e-transferred value is 3.5. Moreover, the percentage of the 2e-pathway is 75% for CONC-700A600 with a total e-transferred value of 2.5 if only the 4e- or 2e-pathway ORR occur during reduction.

#### 3.9.2. Single-Cell Testing

**Figure 10:**
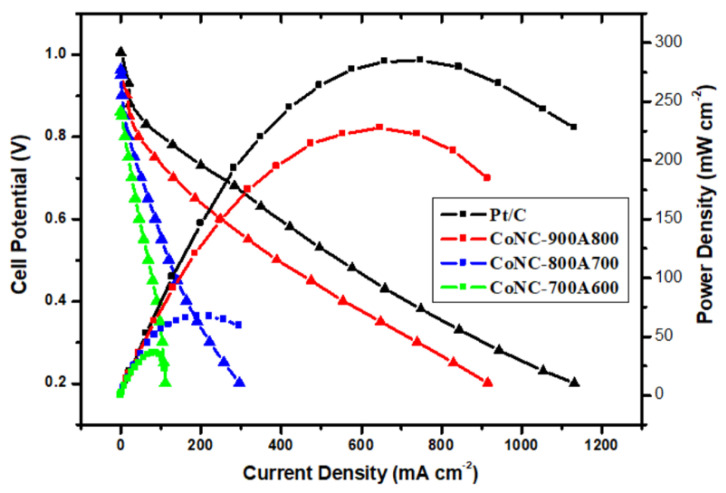
I-V polarization curves and power density of various Co–N–C cathode catalysts.

The Co–N–C based catalyst inks were prepared and coated on the cathode side of the X37-50RT membrane. An MEA was made after coating the anode side with Pt/C ink and hot-pressed. The MEA was then inserted in the cell for single-cell testing. The polarization and power density curves of the single cells with Pt/C or the Co–N–C cathode were obtained from the testing and are illustrated in Figure 10. The cell potential of both CoNC-800A700 and -700A600 decay very fast, resulting from the fast loss of ORR activity of cathode catalysts. The cell potential of CoNC-900A800 can extend to ca. 1000 mAcm^−2^, comparable to that of Pt/C, as seen in Figure 8. Eventually, the max. power densities (P_max_) of CoNC-800A700 and -700A600 (Table 4) are less than 100 mWcm^−2^ (67.0 and 35.8 mWcm^−2^, respectively) due to the 25% and 75% of the ORR following the 2e-pathway. The single cell with the CoNC-900A800 cathode demonstrates a relatively high P_max_ of 227.7 mWcm^−2^ (Figure 10 and Table 5) that is comparable to that made of the Pt/C cathode (285.3 mWcm^−2^) and is higher than that of the common Co–N–C-based AEMFC that used only Pt/C as the anode [47,48,49,50,51,52,53] instead of using expensive Pt-Ru/C as the anode [47,54,55].

## 4. Conclusions

A pyridine-containing polyurea (PUr) was successfully prepared via polycondensation between 2,6 diaminopyridine and MDI with or without Co(II) ions. The obtained Co-complexed PUr (Co-PUr) acts as the precursor that converts to the Co–N–C cathode catalyst for the AEMFC after calcination. The obtained Co–N–Cs demonstrate a porous surface with many micropores created on the surface. Cobalt is presented mainly in crystallized metal and the Co-Nx unit with covalent bonding of nitrogen. The carbon matrix of Co–N–C is mostly made of carbon nanobelts (CNB) or carbon nanoring (CNR), which are possibly grown from the Co seeds. The X-ray diffraction confirms the presence of C(002), Co(111), and Co(200) planes, contributed by either CNB or CNR. The Raman spectra indicate that the carbon structures of Co–N–Cs becomes more and more ordered with increasing calcination temperatures. The non-precious catalyst of CoNC-900A800 demonstrates comparable electrochemical properties related to ORR as commercial Pt/C catalysts such as high on-set and half-wave voltages, high limited reduction current density, smaller Tafel slope, and a high e-transferred number of four. The single cell with the CoNC-900A800 cathode catalyst reveals a Pmax greater than 200 mWcm^−2^, which is better than that of most non-precious cathode catalysts.

In the future, we plan to develop metal-free, nitrogen-containing catalysts from N polymers, considering the possible environmental pollution of Co(II).

## Figures and Tables

**Table 1:** Comparison of I_D_/I_G_ of various cathode catalysts.

CoNC-	I_D_/I_G_ ^a^
700A600	1.04
800A700	1.00
900A800	0.99

^a^ Obtained from Figure 6.

**Table 2:** Compositions of various Co–N–Cs obtained from EDs spectra.

CoNC-	C	N	O	Co
700A600	67.10	19.80	12.21	0.80
800A700	83.80	8.12	6.85	1.23
900A800	83.80	9.30	5.10	1.80

**Table 3:** BET surface area and average pore sizes of various Co–N–Cs.

Co–N–Cs	BET Surface Area (m^2^g^−1^) ^a^	BJH Adsorption Average Pore Diameter (nm) ^b^
700A600	177.74	4.17
800A700	369.43	5.24
900A800	447.81	6.18

^a^: obtained from Figure 8a; ^b^: obtained from Figure 8b.

**Table 4:** Comparison of various electrochemical properties of Co–N–C catalysts.

Catalysts (Pt/C & CoNC-)	On-Set Voltage (V) ^a^	Half-Wave Voltage (V) ^b^	LRCD (mAcm^−2^) ^c^	Tafel Slope (mVdec^−1^) ^d^	# of e-Transferred ^e^	P_max_ ^f^
700A600	0.78	0.39	−2.58	164.1	2.5	35.8
800A700	0.85	0.41	−3.02	165.9	3.5	67.0
900A800	0.88	0.82	−5.10	66.2	4.0	227.7
Pt/C	0.89	0.83	−5.26	67.9	-	285.3

^a^: obtained from Figure 9b; ^b^: obtained from Figure 9b; ^c^: obtained from Figure 9b; ^d^: obtained from Figure 9c; ^e^: obtained from Figure 9d; ^f^: obtained from Figure 10.

**Table 5:** Performance of various Co-containing cathode catalysts of AEMFC.

Cathode Catalyst	Cathode Loading (mgcm^−2^)	Anode Catalyst	Power Density (mWcm^−2^)	Ref.
Co–N–C	0.8	20 wt% Pt/C	228	This work
Pt/C	0.8	20 wt% Pt/C	285	This work
Co–N–C	2.0	60 wt% Pt/C	182	[47]
N-dopes CDC/CNT	0.4	40 wt% Pt/C	310	[48]
ZIF-CB-700	0.4	60 wt% Pt/C	122	[49]
Co/N/MWCNT	0.6	40 wt% Pt/C	115	[50]
Co/N/MWCNT	0.6	40 wt% Pt/C	115	[50]
Co/N/CDC	0.8	60 wt% Pt/C	78	[51]
Co-Fe_3_O_4_/C	0.8	Pt/C	114	[52]
Co–N–C	0.4	60% Pt/C	181	[53]
Co–N–CDC/CNT	0.4	Pt-Ru/C (wt% 50/25/25)	577	[47]
FeCoNC-at	2.0	Pt-Ru/C (wt% 50/25/25)	415	[54]
CoFe_2_O_4_ on Vulcan XC-72	2.4	Pt-Ru	670	[55]

## Data Availability

Not applicable.
